# Keep Your Mask On: The Benefits of Masking for Behavior and the Contributions of Aging and Disease on Dysfunctional Masking Pathways

**DOI:** 10.3389/fnins.2022.911153

**Published:** 2022-08-09

**Authors:** Andrew J. Gall, Dorela D. Shuboni-Mulligan

**Affiliations:** ^1^Department of Psychology and Neuroscience Program, Hope College, Holland, MI, United States; ^2^Neuro-Oncology Branch, National Cancer Institute, National Institutes of Health, Bethesda, MD, United States

**Keywords:** circadian rhythm, sleep, masking, light, dark

## Abstract

Environmental cues (e.g., light-dark cycle) have an immediate and direct effect on behavior, but these cues are also capable of “masking” the expression of the circadian pacemaker, depending on the type of cue presented, the time-of-day when they are presented, and the temporal niche of the organism. Masking is capable of complementing entrainment, the process by which an organism is synchronized to environmental cues, if the cues are presented at an expected or predictable time-of-day, but masking can also disrupt entrainment if the cues are presented at an inappropriate time-of-day. Therefore, masking is independent of but complementary to the biological circadian pacemaker that resides within the brain (i.e., suprachiasmatic nucleus) when exogenous stimuli are presented at predictable times of day. Importantly, environmental cues are capable of either inducing sleep or wakefulness depending on the organism’s temporal niche; therefore, the same presentation of a stimulus can affect behavior quite differently in diurnal vs. nocturnal organisms. There is a growing literature examining the neural mechanisms underlying masking behavior based on the temporal niche of the organism. However, the importance of these mechanisms in governing the daily behaviors of mammals and the possible implications on human health have been gravely overlooked even as modern society enables the manipulation of these environmental cues. Recent publications have demonstrated that the effects of masking weakens significantly with old age resulting in deleterious effects on many behaviors, including sleep and wakefulness. This review will clearly outline the history, definition, and importance of masking, the environmental cues that induce the behavior, the neural mechanisms that drive them, and the possible implications for human health and medicine. New insights about how masking is affected by intrinsically photosensitive retinal ganglion cells, temporal niche, and age will be discussed as each relates to human health. The overarching goals of this review include highlighting the importance of masking in the expression of daily rhythms, elucidating the impact of aging, discussing the relationship between dysfunctional masking behavior and the development of sleep-related disorders, and considering the use of masking as a non-invasive treatment to help treat humans suffering from sleep-related disorders.

## Introduction

### What Is Masking?

Masking allows an organism to respond to changes in exogenous stimuli (e.g., light-dark cycle, social cues, temperature, food, drugs), thereby enabling the organism to act immediately and appropriately (Rietveld et al., 1993); these exogenous stimuli are also capable of “masking” to superpose and integrate with the expression of the endogenous circadian pacemaker, depending on the type of cue presented, the time-of-day when it is presented, and the temporal niche of the organism (Aschoff, 1960, 1988; Wever, 1985; Mrosovsky, 1999; Mrosovsky et al., 1999; Redlin and Mrosovsky, 1999). In this way, masking is capable of complementing and integrating with entrainment if the cues align with arousal and the circadian system [e.g., in diurnal mammals, if lights are on during the day when the suprachiasmatic nucleus (SCN) is active], but can also work independently of the circadian system (e.g., in diurnal mammals, if lights are on at night when the SCN is inactive). In this review, we argue that when exogenous stimuli occur at times that are predictable for the organism, the expression of daily patterns can be strengthened; this is a key point that is often overlooked when considering masking effects on behavior. However, when exogenous stimuli occur at inappropriate times, the expression of daily patterns can be weakened and even misaligned.

The meaning of the term “masking” has evolved over time. When the term masking was first introduced, it was used to describe environmental factors that could prevent a second identity from emerging in an organism. Fry (1947) used the example of humidity being capable of masking the effect of extreme temperature on an organism; high levels of humidity at extreme ambient temperatures can prevent death in an organism, whereas the same extreme ambient temperature with low humidity can result in death. In this way, humidity can act as a “masking factor.” The incorporation of the term “masking” into the circadian literature stemmed from the need to describe the differences in behavior expressed in constant conditions (i.e., endogenous influence) as compared to when an entraining agent is present (i.e., an exogenous, “masking” influence) (Aschoff, 1960). In the early 1980s, these masking effects were described as “noise” that prevented chronobiologists from being able to visualize the endogenous circadian rhythm (Moore-Ede et al., 1982); these masking effects were therefore defined based on their obscuring relationship to circadian rhythms, and the argument at the time was that masking “obscured” the endogenous rhythm that was more important than these exogenous masking influences.

Here we argue that the definition of masking should not necessarily rely on an obscuring nature to the endogenous circadian rhythm, but should rather focus on its direct effects on behavior and physiology which are superposed and integrated with circadian rhythms. In this way, masking can be included as a separate factor in models that predict specific behaviors, such as sleep–wake cycles, to be integrated with circadian rhythms and produce the daily patterns of behavior that are expressed by the organism. Therefore, we argue that masking can be more simply defined today as an acute response to an exogenous stimulus which is superposed on the endogenous circadian rhythm to allow an organism to respond to immediate changes in the environment.

This description of masking as a stimulus-response has been proposed by many (Rietveld et al., 1993; Mrosovsky and Hattar, 2003; Salazar-Juárez et al., 2006; Morin, 2013). As Rietveld et al. (1993) clearly state: “Masking, as is well known, enables an organism to act immediately and in an appropriate way to changes of the environment, integrating with internally produced rhythmicity.” In these definitions of masking, chronobiologists do not necessarily think of masking as something that obscures something more important. Instead, the term masking has evolved to be integrated with circadian rhythms to result in the daily patterning of activity. We have incorporated these new definitions into our proposed definition of masking that considers the law of superposition. By considering masking as a separate factor that allows for superposition to the endogenous circadian rhythm, researchers will be able to experimentally test the power of masking as an independent factor using mathematics and physics to model daily patterns of behavior. There is evidence in the literature to suggest that masking continues to exist in organisms that no longer have an endogenous rhythm (van der Horst et al., 1999; Bunger et al., 2000; Zheng et al., 2001; Papachristos et al., 2011; Pfeffer et al., 2015; Engeland et al., 2018; Yang et al., 2019), providing experimental support for the notion that masking is a separate factor which contributes to daily patterns of behavior. However, the extent to which masking affects the expression of daily rhythms is yet to be determined. For example, we know that masking has time-of-day effects and also differential effects on nocturnal vs. diurnal organisms, but an in-depth examination of how and why masking effects change based on time-of-day is necessary in order to better understand this separate and important masking system, which operates independently but is integrated with endogenous circadian rhythms to result in the daily patterns of behavior that are observed by the organism.

The ability to respond immediately to changes in exogenous stimuli is adaptive to the organism, as it allows the organism to behave in appropriate ways, depending on the organism’s temporal niche (Lu et al., 2010; Bloch et al., 2013; Ghosh et al., 2021). For example, nocturnal (i.e., night-active) organisms mask negatively to powerful exogenous stimuli, such as light, by decreasing activity, and they mask positively to darkness by increasing activity (Aschoff and von Goetz, 1988a; Aschoff, 1999; Shuboni et al., 2012). Diurnal (i.e., day-active) organisms, on the other hand, mask positively to light by increasing activity, and they generally mask negatively to darkness by decreasing activity, although the change in activity in response to darkness is less consistent across organisms as compared to changes in activity in response to light (Aschoff and von Goetz, 1989; Cohen et al., 2010; Shuboni et al., 2012; Barak and Kronfeld-Schor, 2013; Langel et al., 2014). Masking has been tested in many organisms experimentally by providing light or dark pulses of varying intensity and duration during the organism’s subjective night or day, respectively (Salazar-Juárez et al., 2006; Morin and Studholme, 2009, 2014; Shuboni et al., 2012). Importantly, as described above, masking is not only an immediate and acute response to changes in exogenous stimuli but may also result in a disruption to the daily patterns of the organism and the effects can extend for far longer than the pulse itself (Morin and Studholme, 2009). In fact, dim light at night induces masking which, in turn, disrupts the expression of circadian rhythms due to the unexpected change in light intensity for the organism (Frank et al., 2010). It is important to recognize that light is a powerful exogenous stimulus which is capable of resulting in masking behavior, but other exogenous stimuli (as described in the sections below) are also able to mask behavior. We should also point out that masking effects differ depending on which time-of-day (i.e., circadian phase) they are presented to the organism (Aschoff and von Goetz, 1988a,b; Shuboni et al., 2015). In this way, masking and circadian rhythms may bidirectionally influence and complement each other functioning synergistically. Therefore, masking is independent of but complementary to the circadian system.

Masking is important and adaptive for an organism because it allows the organism to respond quickly to sudden, inappropriate changes in the environment. In this way, the masking system is capable of overriding the internal circadian rhythm so that the organism can escape danger. In fact, masking behavior has recently been shown to be evolutionarily conserved in *Drosophila melanogaster* (Ghosh et al., 2021), providing further evidence that masking provides an adaptive advantage for the organism. Importantly, masking can also be complementary to entrainment when the environmental changes are predictable. For example, when diurnal organisms experience light during the day and darkness at night, this predictable change in light intensity aligns with the organism’s active and rest phases, which also align closely with increases and decreases in neural activity of the master clock, the SCN (Ramkisoensing and Meijer, 2015). In contrast to diurnal organisms, nocturnal organisms exhibit an inverted phase relationship between the active and rest phases with electrical activity in the SCN (Brown and Piggins, 2007; Colwell, 2011). Once again, we should recognize that many environmental factors are capable of affecting masking and circadian rhythms, including light, darkness, socialization, temperature, food, and even drug usage (Aschoff, 1999; Kosobud et al., 2007; Hasler et al., 2012; Refinetti, 2015; Fernandes et al., 2021). When these environmental stimuli occur at naturally appropriate and predictable time points, alignment occurs, and masking promotes activity during the same time as signaled by the circadian pacemaker strengthening entrainment. However, when these environmental stimuli are misaligned with behavior (i.e., they occur at inappropriate time points that are not naturally occurring), they instead can modulate, disrupt, or further shift the expression of circadian rhythms (Gronfier et al., 2007). Researchers should take note of the consequences of how these environmental stimuli can either complement or disrupt the expression of daily patterns, especially because exposure to environmental stimuli at night is so prevalent in our society (e.g., increased use of light-emitting screens at night; Chang et al., 2015) which can affect behavior in profound ways (Fonken and Nelson, 2014). For example, exposure to environmental stimuli can mask and therefore significantly complement or disrupt activity levels, sleep, wakefulness, mood, and even quality of life, depending on the time-of-day when the environmental stimulus is presented.

Masking is an important topic to understand within the field of biological rhythms because it is increasingly affecting human health (e.g., increased artificial light exposure at night, decreased natural light exposure during the day, increased use of melatonin) (Hölker et al., 2010; Wright et al., 2013; Kyba and Kantermann, 2016; Li et al., 2022). Thus, masking stands to have significant *deleterious* effects on human health when these exogenous stimuli occur at an inappropriate time-of-day, whereas masking is capable of having significant *beneficial* effects on human health when these stimuli occur at an appropriate or expected time-of-day that align with other exogenous arousal-promoting or sleep-promoting stimuli.

In this review, we have updated Mrosovsky’s (1999) review, which focused on masking, to include new insights, including (1) exploring often overlooked exogenous stimuli that affect masking behavior (e.g., social cues, temperature, food, and drugs), (2) updating the model regarding homeostatic and circadian effects on sleep to include masking effects (Borbély, 1982; Borbély et al., 2016), (3) discussing the neural mechanisms underlying masking in nocturnal and diurnal species, and (4) including new findings regarding the deleterious along with the beneficial impacts that masking can have on human health and behavior (e.g., sleep) as we age.

## Exogenous Stimuli That Affect Masking Behavior

### Light

A zeitgeber is defined as a time-giver and a cue that is capable of entraining circadian rhythms (Golombek and Rosenstein, 2010). Some zeitgebers, such as light, are also capable of masking circadian rhythms; these exogenous stimuli are capable of complementing circadian rhythms either via entrainment, masking, or both, depending on when they are presented to the organism. Light is the most powerful zeitgeber which also acts as a masking stimulus and has a profound effect on behavior of the organism (Aschoff, 1999). The brain is capable of receiving direct input about light via retinal projections, including the retinohypothalamic tract (RHT; Hattar et al., 2006). Melanopsin is a photopigment located in intrinsically photosensitive retinal ganglion cells (ipRGCs) (Beaulé et al., 2003) which project to retinorecipient brain areas to modulate non-image forming vision (Hattar et al., 2006) including entrainment of the master clock to the light-dark cycle, the pupillary light reflex (PLR), and masking to light in nocturnal mice (Hatori et al., 2008). In diurnal Nile grass rats, it has been recently shown that these melanopsin-containing ipRGCs are resistant to excitotoxic injury and are capable of maintaining functional non-image forming behaviors (Fogo et al., 2019). One of the retinorecipient brain areas that receives direct input from these ipRGCs is the SCN, the master clock in mammals; brain areas downstream of the SCN also receive direct input from light (Berson et al., 2002; Warren et al., 2003; Dacey et al., 2005). When the SCN is lesioned experimentally in nocturnal rodents, a multitude of physiological and behavioral outputs become arrhythmic due to the master clock becoming dysfunctional and unable to orchestrate behavioral output (Moore and Eichler, 1972; Stephan and Zucker, 1972).

There has been much debate about whether or not the SCN contributes to masking, given its important role for modulating circadian rhythms (reviewed in Morin, 2013). Redlin and Mrosovsky (1999) have demonstrated that masking to light persists after the SCN is lesioned experimentally in nocturnal hamsters, whereas Li et al. (2005) demonstrated quite the opposite by showing that masking to light is not possible in hamsters following SCN lesions. While this debate has not been settled, and more work needs to be done to determine which hypothesis is correct, evidence from diurnal Nile grass rats (*Arvicanthis niloticus*; Gall et al., 2016) supports data of Redlin and Mrosovsky (1999). It is important to develop sensitive measures to detect the presence of masking and to develop sophisticated and thoughtful methodological techniques to determine whether or not masking persists. On the other hand, one criticism of these kinds of lesion studies is that it is difficult to destroy all SCN cells without causing any damage to nearby areas within the hypothalamus and the retinal fibers that project downstream, which further complicates the findings when this is done. In addition, hypothalamic cells are important for regulating many vital functions of the organism, so if too many neurons are destroyed, death of the organism can result (Gall et al., 2016), further complicating lesion studies. One reason that masking may be able to persist even after significant cell death of the SCN is that there are other retinorecipient brain areas that receive direct light input, including the intergeniculate leaflet (IGL), lateral geniculate nucleus (LGN), olivary pretectal nucleus (OPT), and superior colliculus (SC), which have been shown to be heavily involved in masking to light in nocturnal and diurnal organisms (Morin and Allen, 2005; Gall et al., 2013, 2016, 2017, 2020; Shuboni-Mulligan et al., 2019). These neural connections and their involvement in the process of masking are outlined in a later section of this review (see “Neural mechanisms underlying masking”).

In nocturnal organisms, light pulses are capable of *suppressing* activity in very significant ways (Wever, 1989; Aschoff, 1999). On the other hand, in diurnal organisms, light pulses are capable of *stimulating* activity in very significant ways (Shuboni et al., 2012; Gall et al., 2013; Bonmati-Carrion et al., 2017). Therefore, light is a powerful environmental stimulus which is capable of affecting behavior in immediate and profound ways such that organisms mask to light readily. Importantly, masking to light has been shown to be adaptive for species such as golden spiny mice which may have been essential for these organisms to move into and occupy a diurnal niche (Cohen et al., 2010).

### Darkness

Darkness is defined as the absence of light. Therefore, it could be hypothesized that darkness would result in masking effects that are opposite to the effects of light. Researchers have shown that this is not always the case in both nocturnal and diurnal organisms. Specifically, darkness has less profound masking effects as compared to light in both nocturnal (Aschoff and von Goetz, 1988a; Tsai et al., 2009; Shuboni et al., 2012) and diurnal (Shuboni et al., 2012; Gall et al., 2013, 2017; Langel et al., 2014) species. It is suggested that light is a more powerful stimulus than darkness. For nocturnal organisms that are typically asleep in the light, it would be difficult to respond to a dark pulse while already asleep–a criticism researchers addressed by adding gentle handling to the lights-on condition (Mistlberger et al., 2002). However, this explanation cannot be applied to diurnal organisms since they are typically awake in the light. Current experimental evidence suggests that light is a more powerful stimulus than darkness.

### Social Cues

Social cues are less powerful than light or darkness in both nocturnal and diurnal organisms and are therefore considered weak zeitgebers. In fact, many studies have demonstrated minimal masking effects of social cues in nocturnal (Aschoff and von Goetz, 1988a,b) or diurnal (Castillo-Ruiz et al., 2018) species. In diurnal grass rats under constant conditions, circadian rhythms among grass rats housed as pairs together did not synchronize, whereas ultradian rhythms were capable of synchronizing, suggesting that masking effects of social cues are far less powerful than light-dark cues (Castillo-Ruiz et al., 2018).

Congenitally blind humans who are not capable of receiving light input have been shown consistently to struggle with entrainment, even when they engage in socialization in a predictable way that attempts to align with their circadian rhythm. In fact, blind humans are at a significantly higher risk of being diagnosed with non-24 disorder (Quera Salva et al., 2017), suggesting that without the powerful influence of light, entrainment is challenging. Socialization can certainly mask circadian rhythms, as one can override the circadian system by engaging in activity that aligns with others, but it appears that socialization has a very weak effect on the circadian system, so it becomes very difficult for blind individuals to have a predictable rhythm that is aligned with other zeitgebers, especially when the individual cannot detect changes in light or darkness. Therefore, social isolation in humans is likely to occur due to a rhythm that is free-running and misaligned with exogenous stimuli. Social isolation can have deleterious impacts on behavior. For example, when other social mammals, such as diurnal degus (Lee, 2004), become socially isolated, they exhibit dysfunctional emotional behavior which can be mitigated by an hour a day of resocialization (Braun et al., 2003; Colonnello et al., 2011; Rivera et al., 2021). The free-running nature of the circadian rhythm in congenitally blind humans may lead to depression due to social isolation that occurs due to being active at times when others are sleeping. Although social cues appear to be weak zeitgebers, they have been shown to help re-entrain diurnal degus following phase advances of the light-dark cycle, suggesting that there are clear benefits of socialization on the circadian system (Jechura et al., 2006).

### Temperature

Similar to social zeitgebers, ambient temperature appears to be a weak zeitgeber of the master clock in some species. Temperature tends to align with an organism’s circadian rhythm due to natural fluctuations in ambient temperature that align with the light-dark cycle. Modern heating and air conditioning systems have allowed humans to manipulate this weak zeitgeber, which has been shown to negatively influence the expression of circadian rhythms, especially if temperature does not follow the natural cycle. Specifically, when ambient temperature is set to be significantly warmer during the day and cooler at night, this can serve as a masking stimulus by increasing time spent in rapid eye movement sleep (for review, see Okamoto-Mizuno and Mizuno, 2012). Nocturnal rodents, such as flying squirrels, exhibit masking behavior to temperature without entraining to it (DeCoursey, 1960), providing supporting evidence that temperature can serve as a masking stimulus, but is much less likely to serve as a strong zeitgeber to entrain circadian rhythms. In diurnal rodents, such as degus, temperature cycles are capable of triggering nocturnalism, thereby acting as a non-photic stimulus and impacting temporal niche preference (Vivanco et al., 2010). Although temperature may not be as potent of a zeitgeber as light which acts directly on the SCN, the master clock, temperature is capable of entraining peripheral clocks in mammals (Buhr et al., 2010). On the other hand, when ambient temperature remains constant throughout the day and night, or worse, if the temperature is set to increase at night when humans are sleeping, this can be detrimental to circadian rhythms (for review, see Okamoto-Mizuno and Mizuno, 2012). Temperature provides another case where when aligned with circadian rhythms and when avoiding extreme temperatures, the masking effect can strengthen daily patterns, but when misaligned, the masking effect can weaken daily patterns. It is important for all environmental cues to align with the circadian system whenever possible.

### Food

Food intake also appears to be a weak zeitgeber of the master clock in mammals. This is especially demonstrated when animals eat at an inappropriate time-of-day. For nocturnal organisms, if food is only presented during the day when lights are on and the organism is normally sleeping, this can disrupt sleep-wake patterns such that the organism becomes active during the day so that they can receive nourishment (Refinetti, 2015). When traditionally nocturnal organisms (i.e., mice) are challenged by hunger (or cold) using a work for food (WFF) protocol combined with altered ambient temperatures, hormonal, physiological, and behavioral rhythms are affected in significant ways, including inducing diurnality, without affecting the SCN (van der Vinne et al., 2014). In contrast, other studies have detected effects of caloric restriction on the expression of PER1 and vasopressin in the SCN in mice (Mendoza et al., 2007; Sen et al., 2017), or glucose shortage on the dorsomedial oscillator in the SCN in rats (Yang et al., 2017). Importantly, food restriction to the inactive phase in nocturnal mice resulted in phase shifts to peripheral tissue clocks of the liver, pancreas, heart, and kidney, but the central clock (i.e., the SCN) was not affected, suggesting that feeding time is capable of entraining other clocks in the body, which have been described as food-entrainable oscillators (Damiola et al., 2000; Escobar et al., 2009; Pickel and Sung, 2020). In humans, it has been demonstrated that midnight snacking can weaken daily patterns due to the misalignment of eating and digestion with activity patterns and neural activity of the SCN (Loh et al., 2015). When humans exhibit inappropriate and erratic eating patterns, body weight, metabolism, and sleep patterns can be negatively impacted (Gill and Panda, 2015). Therefore, it is important that food intake is aligned with the active phase of the organism, which ideally should align with other exogenous stimuli such as the light-dark cycle (e.g., eating during the day for diurnal organisms) in order to strengthen daily patterns.

Limited evidence suggests that in addition to the food-driven timekeeping system of peripheral tissues, food is capable of providing a cue which may result in masking. In nocturnal rats that were restricted to food access for 3 h during the lights-on (inactive) phase, the Midline-Estimating Statistic of the Rhythm (MESOR) and amplitude of temperature, heart rate, and locomotor activity were attenuated during food restriction, which the authors interpreted as a masking effect of food restriction (Boulamery-Velly et al., 2005). In addition, Tanaka et al. (1999) provided restricted food access for 6 h during the lights-on (inactive) phase in nocturnal rats and found that the organisms became more active. It should be noted that a recent study showed that heart rate variability (HRV) and blood pressure (BP) are directly affected by changes in blood glucose levels which rise as a result of food intake, and that various types of carbohydrates affect heart rate differently, suggesting that acute changes in food intake affect physiology in humans (Eckstein et al., 2022). There is also evidence in the literature suggesting acute meal-induced metabolic changes result in acute inflammatory responses in humans that may lead to an increased risk of chronic inflammatory diseases (Mazidi et al., 2021). Altogether, these studies point to the potential role food may play as a masking stimulus; more work needs to be done under constant conditions in order to better understand the effect food has on masking behavior.

### Drugs

Within this section we will examine a sample of over-the-counter drugs for self-medication and prescribed drugs for medical purposes that can function as masking agents; this section is not an exhaustive list of drugs or their effects on behaviors (e.g., sleep, activity patterns, arousal).

Melatonin is an endogenous hormone released primarily by the pineal gland, but is also synthesized in retina and other tissues (Hardeland, 2017). The release of melatonin follows a circadian rhythm and begins to rise at the beginning of the dark phase in both diurnal and nocturnal mammals. Importantly, the endogenous release of melatonin can be inhibited or masked by light stimulation in both diurnal and nocturnal mammals so that production is suppressed when light is presented at night (Kennaway et al., 2002; Claustrat et al., 2005). As society increases nighttime light usage through environmental lights and electronic light-emitting devices, melatonin levels are being inhibited at a time that they should be secreted at the highest levels. When melatonin levels are inhibited in humans, it results in a disruption in sleep timing such that sleep onset is significantly delayed (Zeitzer et al., 2000). One way to overcome this delay in sleep onset is to turn electrical lights off and keep screens off at night as darkness starts to naturally occur. Another way to overcome this reduction in melatonin levels is to take an exogenous form of melatonin, often prescribed by physicians for their patients to fall asleep faster. This exogenous melatonin has a direct hypnotic effect that can induce masking of sleep in both humans and primates (Zhdanova, 2005). These effects, however, are not the same in nocturnal rodents who do not show the same sleep-inducing effect (Sugden, 1983; Fisher and Sugden, 2010; Barbosa-Méndez and Salazar-Juarez, 2020) with most laboratory mouse strains also not producing circulating melatonin (Kennaway, 2019). It is important to note, however, that while melatonin deficiencies are possible in humans (Hardeland, 2012), these should be diagnosed by a physician. Individuals that do not have a melatonin deficiency should first consider non-invasive treatments such as removing the light sources at night which prevent the melatonin rise necessary to help them fall asleep naturally. Melatonin administration is yet another case where when taken properly and at the appropriate time of day, it can serve as a zeitgeber to entrain the endogenous clock, and it can also result in masking behavior to induce sleepiness. However, when melatonin is taken in the afternoon, phase shifting can result (Crowley and Eastman, 2013), making it more difficult for individuals to stay asleep at night, resulting in the opposite effect that the consumer was hoping to alleviate.

Marijuana is also an exogenous substance that is often used to help users fall asleep faster. While it has been shown that marijuana is capable of allowing organisms to fall asleep faster (Gates et al., 2014), we should also acknowledge that it likely has masking effects which will in turn affect the circadian system. Less is known about these effects, but we hypothesize that if marijuana is taken at random times using random dosages, similar to melatonin, sleep will be elicited, but circadian desynchrony will result. On the other hand, if marijuana is taken chronically at the same time each night using the same dosage in humans, circadian synchronization can result, making it more likely that the individual will fall asleep and stay asleep throughout the night (Whitehurst et al., 2015). Of course, doing so is likely to result in dependency on the drug, and therefore when the drug is not taken, withdrawal effects (Budney et al., 1999) and potential disruptions to the circadian system are likely to result. More work needs to be done to elucidate the effects of cannabis usage on the masking and circadian systems.

Alcohol is another exogenous substance that in the population is commonly used to self-medicate for insomnia (Goodhines et al., 2019). Consumption of alcohol before sleep can directly impact body temperature (Kleitman, 1939); these masking effects on body temperature have been shown to impact not only homeostatic sleep, but also the circadian system (Danel et al., 2001). Alcohol directly impacts regions of the brain that are responsible for homeostatic sleep and alter the expression of sleep architecture (Williams and Salamy, 1972). Alcohol also affects entrainment of the SCN to photic and non-photic zeitgebers (Ruby et al., 2009; Prosser and Glass, 2015) and impacts the expression of endogenous rhythms in constant conditions (Ruby et al., 2017). Injections of ethanol in mice can also have direct effects on peripheral clocks; in the skeletal muscle and liver, injections changed the expression of core clock genes (Tice et al., 2021), However, the relationship between sleep and alcohol consumption is problematic as alcoholism is also linked to the development of sleep disturbances (Stein and Friedmann, 2006). Chronic alcohol use can directly impact the homeostatic sleep response, wildly altering the expression of sleep architecture (Brower, 2001; Thakkar et al., 2015) and disrupting responses to sleep challenge (Armitage et al., 2012). The use of alcohol as a sleep aid should therefore be considered with great caution as the switch between casual and chronic use could exacerbate the incidence of sleep disorders. Clearly, there are immediate masking effects of alcohol on sleep and impacts on the homeostatic and circadian system in the short-term with further detrimental effects on both systems with chronic use. The alcohol literature is vast for both homeostatic and circadian effects, however, the role of masking remains unclear (O’Boyle et al., 1995) and deserves further exploration.

Prescription sleep aids can also be used to treat sleep disorders in patients (Lie et al., 2015). The United States Food and Drug Administration (FDA) has approved the following agents for the treatment of sleep problems: benzodiazepine receptor agonists (benzodiazepines: e.g., Halcion, Prosom, Restoril, and Doral); non-benzodiazepines (e.g., Zolpidem and Zaleplon), melatonin receptor agonists (e.g., Ramelteon), and orexin receptor antagonists (e.g., Suvorexant). These medications are designed to help alleviate the effects of chronic sleep disorders that are prevalent in the population and become more common with age (Ram et al., 2010; Partinen, 2011). The mechanism of action for each drug is unique but can have immediate masking effects with consumption. Benzodiazepines have acute sedative and anxiolytic effects (Konopka and Zimmerman, 2014). Taken acutely or chronically, these agents also impact the expression of the homeostatic sleep system, altering sleep architecture (Holbrook et al., 2001; Ribeiro de Mendonça et al., 2021). Within the circadian system, benzodiazepines can produce major shifts in the endogenous rhythm (Turek and Van Reeth, 1988) and can also impact entrainment to light (Van Reeth and Turek, 1987). Within the U.S., 12.5% of the population use benzodiazepines and within this group, 17.1% misuse the drug in many cases to help further treat sleep issues (Blanco et al., 2018). Like with alcohol, the use of these substances should be closely monitored by a physician and the understanding of long-term impacts on sleep considered. Again, these agents provide a framework to appreciate the complex relationship between masking, homeostatic, and circadian systems and should be further explored in this context.

In contrast to the agents discussed above, some drugs can function to promote activity rather than sleep. Stimulants increase the activity of the brain and can be prescribed for disorders, like ADHD, or used recreationally, such as cocaine, MDMA, or caffeine (Luethi and Liechti, 2020). Caffeine is consumed daily by a majority of the population, with 85% of US adults having at least one caffeinated beverage per day (Mitchell et al., 2014). Consumption of caffeine can induce an immediate thermogenic effect with an increase in body temperature and metabolism (Wager-Srdar et al., 1983; Koot and Deurenberg, 1995). Locomotor activity is also induced 30 min after injection of 0.5–16 mg/kg caffeine in mice (Kayir and Uzbay, 2004). Clearly, caffeine can have a masking effect on several different behaviors, but again circadian rhythms and homeostatic sleep are also directly impacted by the drug. Caffeine can impact the endogenous rhythm (Oike et al., 2011; Burke et al., 2015) and also alter the response of the SCN to light entrainment (Ruby et al., 2018). For homeostatic sleep, caffeine has been shown to attenuate the build-up of sleep propensity during the waking hours (Landolt et al., 2004). As with other drugs however, caffeine dependency can also lead to sleep disruption and daytime sleepiness (Roehrs and Roth, 2008). Stimulants, like drugs designed to induce sleep, should be consumed with the understanding that issues with chronic use are possible and likely.

Any exogenous substance (e.g., melatonin, marijuana, other drugs) that reliably affects behavior and is taken chronically has the capability of resulting in masking effects which may affect the circadian and homeostatic systems. When these drugs result in phase advancement, phase delay, or misalignment with circadian rhythms, consequences such as sleep disruption, changes in mood, altered digestion, altered metabolism, or hormone dysregulation are possible. It is important to recognize that when these substances result in circadian desynchrony, long-term effects can result. On the other hand, when these substances result in circadian synchronization, this can be beneficial, but the side effects of the drug and consequences of withdrawal should be considered.

### Stress

Stress is a physiological response to the presentation of an environmental cue, a condition or agent that disturbs the physical or mental wellbeing of an organism, which can result in adaptive behaviors (Selye, 1950). Stressors presented within the environment directly trigger the autonomic system and the hypothalamic-pituitary-adrenocortical (HPA) axis, which results in elevated levels of plasma glucocorticoids (Ulrich-Lai and Herman, 2009). The activation and expression of stress hormones is an important factor that regulates many of the physiological processes listed above, including social interactions, consumption of food, and the administration of drugs (Ota et al., 2021). Stressors, such as social stressors, have been shown to affect the circadian and masking (non-circadian) systems (Meerlo et al., 2002), but these effects are dependent on the type of stressor presented, suggesting that for social stress, the output behavior is strongly masked, and the central pacemaker is not perturbed. Social cues are considered weak zeitgebers and the relationship between circadian rhythms and masking may be different than other stimuli.

Light, a strong zeitgeber, is also directly linked to the secretion of stress hormone. In nocturnal rodents, light induced increases in corticosterone occur only during the subjective night when levels of stress hormone are the highest for animals (Ishida et al., 2005; Mohawk et al., 2007). Ablation of the SCN in these animals negatively impacted the daily day-night differences in the secretion of the hormone, indicating that the SCN is critical for light induction of the adrenal gland. However, we should be cautious with interpretations of these findings, as lesions of the SCN have been shown to also impact the projection of retinal fibers downstream. For animals with ablated circadian clock genes, Bmal1 KO mice maintained daily patterns in stress hormone under LD cycles, indicating that functional clocks are not required for the daily patterns (Engeland et al., 2018). The role of these neural structures in light responses to the secretion of stress hormone should be further explored to better understand how light is capable of acting as a stressor to affect masking behavior.

When comparing expression of stress hormones between temporal niches, there are significant differences in the general profile of stress hormones between nocturnal and diurnal organisms, with peak levels secreted during the day for diurnal animals but at night for nocturnal animals (Mohawk and Lee, 2005; Bohák et al., 2013). The data for light exposure and stress hormone levels for diurnal organisms has only been performed in humans and is more inconsistent, with studies finding no changes (Beck-Friis et al., 1985; McIntyre et al., 1992; Leproult et al., 1997, 2001; Thalén et al., 1997; Scheer and Buijs, 1999; Lavoie et al., 2003; Rüger et al., 2006), increases (Figueiro and Rea, 2012; Rahman et al., 2019), and decreases (Kostoglou-Athanassiou et al., 1998; Scheer and Buijs, 1999; Jung et al., 2010) in levels of glucocorticoids with exposure to light. These studies use varying times and light intensities/durations which could impact the response of the adrenal gland (Jung et al., 2010). Indeed, it is important to note that with any masking studies, the duration of the stimulus along with time of presentation should be taken into consideration, underscoring the importance of studies that focus on comparative analyses using careful consideration of these factors. Further testing in other diurnal species would be needed to better understand circadian profile of the masking response to light response in stress hormones; a diurnal rodent species would be easier to control for other variables than human subjects. It should be noted that diurnal grass rats given light at night (LAN) demonstrate alterations in stress hormones (Ikeno et al., 2016).

## Masking Interacts With the Circadian and Homeostatic Systems to Affect Behavior in Profound Ways

### Real-World Consequences of Misaligned Masking: Jet Lag and Shift-Work

Acute pulses or changes in light intensity clearly have effects on behavior in an immediate way. Organisms are capable of responding directly to the change in light intensity, especially when the light is bright and stimulates melanopsin in intrinsically photosensitive retinal ganglion cells (ipRGCs) in the retina which, in turn, stimulate retinorecipient regions of the brain (Beaulé et al., 2003). What happens to behavior when those changes in light intensity misalign with circadian rhythms over longer periods of time? Misalignment occurs when humans engage in shift work. The negative consequences of shift work are especially prevalent and invasive when shift workers attempt to switch back and forth between working (i.e., being active at night) while socializing with friends and family on the days off (i.e., being active during the day). This “misalignment” results in severe consequences for human health (Åkerstedt and Wright, 2009). When we think about shift workers, we often think about essential workers such as nurses, doctors, or factory workers who must stay awake during the night hours in order to keep society safe. This comes at a great cost to the shift workers, especially if they flip-flop from being night-active when working to becoming day-active on their days off (Åkerstedt and Wright, 2009). When this is done, the circadian system is unable to fully adjust to the shift work, and therefore humans are only able to stay awake at night because the masking system is overriding the endogenous circadian rhythm that is misaligned with behavior (Vokac et al., 1981). Nurses that engage in many years of shift work have been shown to be at a higher risk of developing breast cancer (Szkiela et al., 2020), depression (Booker et al., 2020; Park et al., 2020), and anxiety (Booker et al., 2020). Animal models of shift work with nocturnal mice have also revealed the deleterious effects of misaligning circadian rhythms and behavior which occurs due to effects of masking, but that these effects are most severe when the organism is expected to flip-flop their behavior on days of work vs. days of rest, also known as “circadian desynchrony” (Barclay et al., 2012).

It is possible for a shift worker to be less negatively affected if they completely shift their circadian rhythms to being night-active, even on their days off. Of course, it is difficult to become a permanent night worker (less than 3% have a complete circadian adjustment), and this can have negative implications due to fewer social interactions and isolation, so the benefits are few and difficult to achieve (Folkard, 2008). It is also important to recognize that artificial light is far less intense in brightness than natural light (Remé et al., 1991; Wirz-Justice et al., 1996). There are many benefits of receiving light input from the sun to humans, including Vitamin D, benefits to mood, and strengthening of entrainment (Duffy and Wright, 2005; Mead, 2008). There is no good substitute for natural sunlight, so even if a shift worker works at night and is able to shift their circadian rhythms fully to become night-active, they are still missing out on the beneficial effects that sunlight provides. While it is important to emphasize that shift workers are necessary and provide important services to others, it comes at a cost to health. Although some steps can be taken to reduce the negative health effects on these shift workers, such as aligning circadian rhythms with activity on work days and days off, there are still costs to consider such as social isolation and reduced natural light.

Wright et al. (2013) have shown that artificial light at night is harmful to the expression of circadian rhythms in humans, and that natural light exposure during the day can help strengthen entrainment. While it is most beneficial for diurnal organisms to align their behavioral profiles to be active when the sun is out, we recognize that this is not always possible. Therefore, in order for shift workers to have the least amount of deleterious effects, they should continue to have the same sleep–wake schedule on their days off.

### Masking Affects Sleep and Activity Patterns by Interacting With Circadian Rhythms and Homeostasis

**FIGURE 1:**
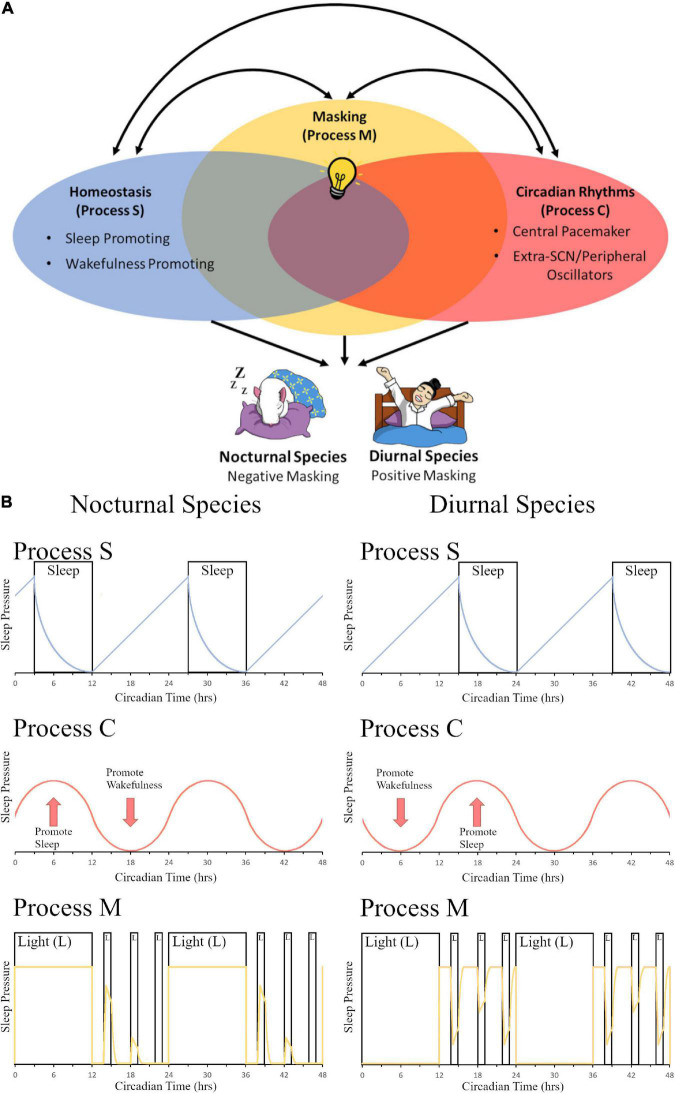
The contribution of light-induced masking to the two-process sleep regulation model. **(A)** In the two-model system of sleep regulation, sleep is driven by the homeostatic process (Process S, Blue Oval) and the circadian process (Process C, Red Oval). Anatomically, these regions can be divided into sleep or wake promoting regions in Process S and Central pacemaker (SCN) or Extra-SCN/peripheral Oscillators in Process C. Here we also propose a third component to the model, masking (Process M, Yellow Oval). In this depiction, the systems share some overlap between all three processes as extra-SCN oscillators, regions of the brain that promote wakefulness/sleep, and masking activation of the brain can all occur within the same region. Additionally, different stimuli that govern sleep and circadian rhythms, such as light (depicted as a light bulb), can induce opposing effects in nocturnal versus diurnal species. Light for a nocturnal rodent induces sleep, causing a negative masking response in activity; while for a diurnal human, light increases arousal and produces a positive masking effect on activity. **(B)** A model of changes in sleep pressure are depicted for nocturnal species (left panel) and diurnal species (right panel). Process S represents the homeostatic system and how sleep pressure builds at night for nocturnal organisms and during the day for diurnal organisms, with sleep pressure dissipation when the organism is sleeping. Sleep pressure peaks at opposite times of day for nocturnal vs. diurnal organisms. Process C represents the circadian system. Sleep pressure peaks at the beginning of the day for nocturnal organisms, whereas it peaks at the beginning of the night for diurnal organisms. Process M represents the masking system. The yellow line depicts sleep pressure, which peaks during the day for nocturnal organisms, and at night for diurnal organisms; light at night is capable of increasing sleep pressure in nocturnal organisms, whereas it is capable of decreasing sleep pressure in diurnal organisms. Panel **(B)** (Process S and Process C) is redrawn from Borbély (1982) and Borbély et al. (2016). Panel **(B)** (Process M) is modeled based on masking data from Shuboni et al. (2015).

Evolutionarily, sleep behavior is found across the animal kingdom and plays an essential role in normal functioning with deprivation leading to severe symptoms including issues with development, cognitive functioning, and overall survival (Miyazaki et al., 2017). A two-process mechanism that regulates sleep function of mammals was first proposed by Borbély (1982). They postulated that a sleep-dependent homeostatic process (Process S) along with a sleep-independent circadian process (Process C) regulated daily variations in sleep propensity, duration, and timing. Since this milestone publication, the authors have updated their model to include new findings about both the processes and have explored the greater level of complexity and interconnectivity between the two systems (Borbély and Achermann, 1999; Borbély et al., 2016). Others have proposed the addition of more variables to this model (Duhart et al., 2021); however, here we propose a streamlined addition of one concise third process to the system–masking behavior (Process M)–which encompasses many different variables listed above that all function via similar immediate behavioral changes and utilize the existing homeostatic and circadian pathways to help regulate sleep and activity depending on temporal niche (Figure 1). While the descriptions of these mechanisms in this section are focused on sleep, the authors would like to propose that 3-process (homeostatic, circadian, and masking) models could be applied to other behaviors described in the review. Our model is based in part on the work of Borbély (1982) and Borbély et al. (2016) which postulate that Process S and Process C interact with each other; Borbély’s model, however, does not consider the effects of masking. Our model is also based in part on the work of Moore-Ede et al. (1982) which postulates that exogenous influences (i.e., masking) are passive components while endogenous oscillations (i.e., circadian rhythms) are active components to contribute to a measurable daily pattern (i.e., an overt rhythm); Moore-Ede’s model, however, does not consider homeostatic influences. We have proposed a model (Figure 1A) to include all three processes in one model (e.g., Process S, Process C, and Process M), and we have included the important influence of chronotype (e.g., nocturnal vs. diurnal species; Figure 1B).

### Circadian Rhythms [Process C]

On Earth the 24-h cycling of light and darkness has led to the development of daily patterns in behavior (Menaker et al., 1997; Bhadra et al., 2017). These behaviors and physiological processes are said to have circadian rhythms when (1) the rhythm persists under constant conditions, (2) can adapt to environmental zeitgebers by entrainment, and (3) are temperature compensated (Pittendrigh and Daan, 1976). In mammals, the neuroanatomic regulation of circadian rhythms was first described with the identification of the suprachiasmatic nucleus of the hypothalamus (SCN) which after ablation eliminated the expression of many behavioral and physiological circadian rhythms (Moore and Eichler, 1972; Stephan and Zucker, 1972). For many years, the SCN was thought of as a master clock controlling circadian rhythms via endogenous patterns originating solely within the nuclei. However, with the discovery of a self-sustained transcription translation feedback loop of clock genes, which are present and cycling beyond the SCN in most tissue and cell types across the body, the idea of peripheral oscillators and their autonomy has become a popular topic of research in the field (Yoo et al., 2004; Mure et al., 2018). The regulation of behaviors like sleep and activity relies on distinct interactions between circadian rhythms from the SCN (central pacemaker) and peripheral oscillators with the masking and homeostatic systems working to produce a final profile of behavior. Till Roenneberg and Merrow (1998) discuss the relationship between the circadian pacemaker and environment cues which they call zeitnehmer, “time takers” that act as both inputs and outputs of the circadian system and function to feedback and strengthen the clock. These zeitnehmers, which includes light, sleep, feeding, and activity (Roenneberg et al., 2013), are masking stimuli and complements the argument made for the interconnection between circadian rhythms, masking and homeostatic sleep.

Circadian rhythms generated in the SCN of an intact animal influence the expression of homeostatic sleep (Deboer, 2009; Guillaumin et al., 2018) and masking (Shuboni et al., 2012) based on time-of-day. When the SCN is ablated, these two systems (Mistlberger et al., 1983; Edgar et al., 1993; Gall et al., 2016) continue to function independent of the central pacemaker; in the case of homeostatic sleep processes, sleep becomes fragmented in constant conditions. Masking maintains a 24 h profile similar to the LD cycle that is presented with the animal only becoming arrhythmic if placed in constant conditions or if the RHT is damaged during lesion, as demonstrated using a diurnal rodent model (Shuboni, 2013; Gall et al., 2016). Importantly, as noted earlier, further experimentation is required to show that the SCN is not required for masking. However, evidence of the dissociation between the circadian and masking system in their responses to light can be observed in the nocturnal California mouse (de Groot and Rusak, 2002), where one animal did not entrain to light but was still capable of negatively masking. A key feature to note in all the discussions of circadian rhythms, homeostatic sleep, and masking is that under natural conditions, daily patterns of light/dark exposure are maintained. Because light is a consistent, rhythmic daily stimulus and all three systems are responsive to their presentation, we hypothesize that brain regions important for regulating homeostatic sleep and extra-SCN circadian rhythms are critical for inducing masking and rely on the behavior to promote timing of sleep and activity. Our work has shown that light directly activates regions of the brain important for the regulation of homeostatic sleep and in our diurnal rodent model these regions have also shown rhythmic daily patterns of activation (Shuboni et al., 2015; Shuboni-Mulligan et al., 2021). Lesions of some of these regions have been shown to have dramatic implications for masking behavior (see Neural mechanisms of underlying masking below).

When examining the impact of clock gene deletion and the relationship between the three processes there are also some interesting findings. Masking behavior persisted in BMAL1 (Bunger et al., 2000; Pfeffer et al., 2015; Yang et al., 2019), PER1/2 (Zheng et al., 2001), and CRY1/2 (van der Horst et al., 1999; Papachristos et al., 2011) mouse knockouts, as each showed daily activity patterns in LD conditions and only became arrhythmic in DD. Yang et al. (2019) further suggested that the lack of circadian rhythmicity and sole dependence on masking improved the health of mice when the deletion was introduced at adulthood and animals were experiencing circadian disruption protocols. Another situation where circadian rhythms are not necessarily advantageous is in animals found around the arctic circle or in deep underwater caves that no longer express circadian rhythms and depend on masking to other cues to govern behavior (Lu et al., 2010; Beale et al., 2016). This does not however remove the possibility that extra-SCN oscillators and clock genes may be independently sensitive to light and strengthen the masking response (Shuboni-Mulligan et al., 2019). Homeostatic sleep, on the other hand, seems to show a direct impact of clock gene knockouts on NREM sleep and other factors (Franken, 2013; Deboer, 2018). A further exploration of clock genes in the circadian visual system and the homeostatic sleep circuitry of the brain in LD and under constant conditions is necessary in order to provide an understanding of these regions with and without SCN mediation. Circadian rhythms are an important mediator of sleep and masking; these three systems work together to result in the complex behavioral phenotype (Figure 1).

### Homeostasis [Process S]

Sleep homeostasis can be conceptualized by monitoring sleep debt; across the wake period, the level of sleep pressure accumulates across time until sleep is initiated, then it decreases during the sleep phase until the subject wakes (Borbély et al., 2016). When the homeostatic system is tested using sleep deprivation protocols, the variable that best quantifies sleep debt is slow-wave activity using EEG measures in humans (Achermann et al., 1993; Borbély, 2001). From the forced desynchrony literature, scientists were able to disentangle the effects of circadian rhythms from the homeostatic sleep process as sleep occurred at different portions of the free running human circadian rhythm (Daan et al., 1984; Dijk and Czeisler, 1995). Above we discussed the impact of circadian rhythms on the expression of homeostatic sleep. Conversely, sleep deprivation can also directly impact the sensitivity to light of the circadian system to phase shifting, caused by a decrease in light sensitivity in nocturnal animals (Mistlberger et al., 1997; van Diepen et al., 2014) or an increase in light sensitivity in diurnal animals (Jha et al., 2017). Sleep deprivation also impacts the expression of circadian clock genes in mice (Husse et al., 2012; Curie et al., 2013), demonstrating the interconnectivity of these systems.

Altogether, the circadian system and the homeostatic system are separate systems with interconnections that influence sleep and wakefulness (Figure 1). In the subsequent sections we will discuss the anatomical regions that are involved in the masking response; both the circadian visual systems and regions of the brain associated with the homeostatic sleep process will be examined in their relationship to masking.

## Neural Mechanisms Underlying Masking

As noted above in the “light” section of this review, light is a powerful zeitgeber that is capable of entraining circadian rhythms. Light enters the visual system by striking intrinsically photosensitive retinal ganglion cells (ipRGCs) which send neural signals to retinorecipient brain areas via the retinohypothalamic tract (RHT) and via the release of glutamate and pituitary adenylate cyclase activating polypeptide (PACAP) from those ipRGCs. Some of these retinorecipient brain areas that have been studied extensively with respect to their masking effects are the IGL, SCN, OPT, and SC.

**FIGURE 2:**
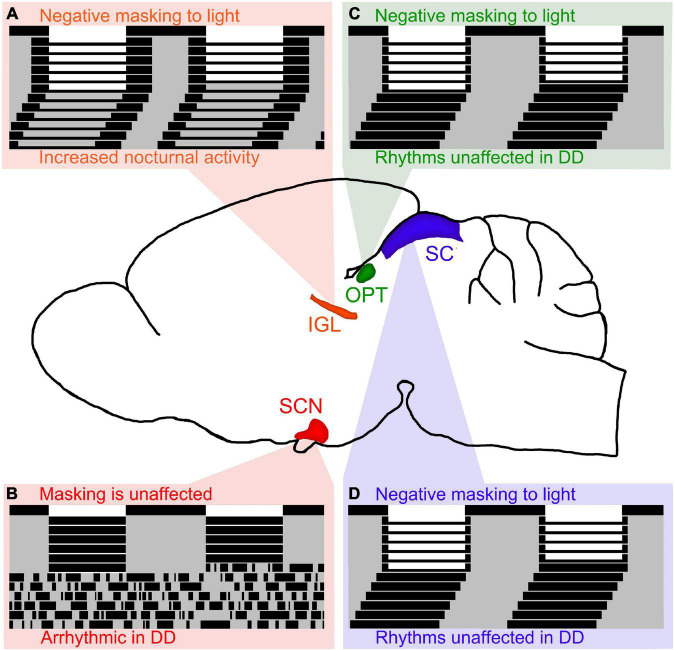
Lesions of retinorecipient brain areas in Nile grass rats, a diurnal species, results in differential effects on masking and circadian rhythms. **(A)** Intergeniculate leaflet (IGL) lesions in Nile grass rats result in negative masking to light and increased nocturnal activity persists in DD (figure redrawn from Gall et al., 2013). Of the 4 retinorecipient brain areas lesioned thus far in Nile grass rats, the IGL is the only brain region that affects both masking and circadian rhythms in significant ways. **(B)** Suprachiasmatic nucleus (SCN) lesions in Nile grass rats result in arrhythmia in constant darkness (DD), but does not affect masking to light (figure redrawn from Gall et al., 2016). **(C)** Olivary pretectal nucleus (OPT) lesions in Nile grass rats result in negative masking to light, but circadian rhythms are unaffected (figure redrawn from Gall et al., 2017). **(D)** Similar to OPT lesions, lesions of the superior colliculus (SC) in Nile grass rats result in negative masking to light, but circadian rhythms are unaffected (figure redrawn from Gall et al., 2020).

The IGL has been shown to play an important role in the expression of circadian rhythms and masking behavior to light in both nocturnal and diurnal rodents. In nocturnal rodents, IGL lesions are capable of enhancing negative masking responses to light (Redlin and Mrosovsky, 1999), and modifying the period of free running rhythms along with altering the phase angle of entrainment (Pickard et al., 1987; Harrington and Rusak, 1988; Johnson et al., 1989; Pickard, 1989). In diurnal rodents, IGL lesions reverse the way these organisms respond to light pulses and result in a night-active phenotype, even in constant conditions (see Figure 2A; Gall et al., 2013). Converging evidence suggests that the IGL is critical for both circadian rhythmicity and the direct behavioral responses to light (i.e., masking).

In nocturnal animals, some reports suggest that the SCN is necessary for masking responses to light, while other reports suggest that it is not. The only report in diurnal species suggests that the SCN is not necessary for masking responses to light (see Figure 2B; Gall et al., 2016). More work needs to be done to elucidate the role of the SCN with respect to masking, but given that multiple reports suggest that the SCN is not required for masking responses to light, we hypothesize that this is the case for nocturnal and diurnal species. Therefore, it is our belief that the SCN is necessary for the expression of circadian rhythms, but not for masking behavior to light.

It is important to note that lesions of the IGL and SCN are technically challenging, especially due to their proximity to image-forming visual pathways, including the lateral geniculate nucleus (LGN) which lies immediately dorsal and ventral to the IGL, and the optic chiasm which lies immediately ventral to the SCN. As Hatori et al. (2008) have shown, melanopsin-containing ipRGCs project to non-image forming brain areas, and are critical for entrainment, the pupillary light reflex, and masking behavior. In the studies we did using diurnal Nile grass rats, we were careful to note that in cases that included extensive damage to these nearby areas involved in image-forming vision, we observed effects that were inconsistent with damage to only the IGL or SCN. For example, when the LGN was damaged but the IGL was intact, we did not observe the effects on masking or circadian rhythmicity that we observed when the IGL was damaged. When the optic chiasm was damaged but the SCN was intact, we observed free-running behavior, which was not observed when the SCN was damaged alone. Therefore, based upon the available evidence, it appears that the IGL and SCN play important roles in non-image forming vision (e.g., masking and entrainment) that cannot be explained by damage to image-forming brain areas (e.g., LGN, optic chiasm).

The OPT and SC are both midbrain structures that have been shown to play an important role in masking to light, but not for the expression of circadian rhythms. In nocturnal organisms, the OPT and SC have been shown to be involved in mediating behavioral responses (e.g., sleep) in response to changes in illumination (Miller et al., 1998, 1999; Zhang et al., 2019). In diurnal organisms, when the OPT and SC are lesioned, masking behavior to light is disrupted, but circadian rhythms are not (see Figures 2C,D; Gall et al., 2017, 2020).

Altogether, it is clear that several brain areas are involved in masking effects to light (e.g., IGL, OPT, SC), whereas only the SCN and IGL appear to affect circadian rhythms in significant ways. We hypothesize that retinorecipient thalamic brain regions, such as the IGL, are the only ones capable of modulating *both* circadian rhythms and masking.

Beyond the circadian visual system, our work also demonstrated that other brain regions important for homeostatic sleep and wakefulness (Saper et al., 2005) are also stimulated via light exposure during masking responses (Shuboni et al., 2015). These regions include the lateral hypothalamus (LH), dorsal raphe (DR), locus coeruleus (LC), and ventrolateral preoptic area (VLPO), which all exhibited higher levels of cFOS labeling after light exposure in diurnal grass rats but not nocturnal mice. Activation of these sleep regions by light may be important in regulating issues with mood (Bedrosian and Nelson, 2017). A diurnal model of seasonal affective disorder (SAD), a mood disorder that leads to depressive episodes during the winter months, involves an orexin-DR pathway that is responsive to light and regulates mood in grass rats (Adidharma et al., 2012, 2019; Deats et al., 2014). Taken together, light exposure in diurnal mammals is capable of stimulating arousal and improving mood via activation of these brain areas that are part of the masking system.

## Sleep Disturbances and Masking

Sleep disturbances are a common occurrence in the healthy population (Bliwise et al., 1992; Lindberg et al., 1997; Essien et al., 2018) and become more prevalent with illness (Altman et al., 2017; Pisani and D’Ambrosio, 2020), increasing the severity of other symptoms (Irwin et al., 2013) and impacting the disease trajectory of individuals (Innominato et al., 2015a; Gottfried et al., 2020). In a systematic review of patients that were chronically ill and hospitalized, the most common patient-reported factors which lead to disrupted sleep during care were noise and light (Honarmand et al., 2020). In the hospital setting, the impact of light on patient outcomes has been extensively studied and alterations to the design of facilities to better support healthy sleep have been proposed and successfully implemented (Acosta et al., 2017; Vethe et al., 2021). Light exposure to ill patients has a duality of effects: outcomes improve with more natural light during the day (Park et al., 2018; Lusczek and Knauert, 2021) but light can have detrimental effects if presented at night (Craig and Mathieu, 2018; Albala et al., 2019). Currently the literature lauded the circadian system as the main perpetrator of these risks and benefits; however, masking is also key to promoting these immediate effects of light on sleep. In fact, Hubbard et al. (2021) argue that sustained light in mice can directly affect non-circadian photic regulation to influence behavior in profound ways, especially sleep and wakefulness; they conclude by arguing that the non-circadian system is just as important as the circadian system for influencing behaviors such as the sleep-wake cycle. In fact, their results strongly support our main argument that sustained direct light effects (i.e., masking in the way we define it here in this review) *must* be considered, in addition to the circadian system, to understand the deleterious health consequences of improper timing and amount of light exposure in society. It is also important to note that patients live in a natural environment with a light-dark cycle under which masking, homeostatic sleep, and circadian rhythms all work together in synchrony to promote activity and sleep cycles. When this light-dark cycle becomes disrupted, as it may in a hospital setting where lights are constantly on, patients may suffer from a debt of sleep.

Beyond the environmental influences that arise in the hospital setting, patients that suffer from chronic illness can develop sleep issues associated with their disease or the therapies used for their treatment (Davidson et al., 2002; Berger et al., 2005; Lui and Ancoli-Israel, 2015). These sleep disturbances manifest as different sleep problems (e.g., insomnia, daytime sleepiness), and their causes may vary based on the disease, treatment, and pre-existing sleep disorders (Foley et al., 2004). In oncology, sleep issues can also present as fatigue, a more loosely defined variable described as a feeling of tiredness or weakness (Ancoli-Israel et al., 2001). In other peripheral cancers, treatment-related fatigue is hypothesized to be associated with the activation of proinflammatory cytokines, such as IL-1β and TNF-α (Collado-Hidalgo et al., 2006). The mechanisms by which this inflammation triggers the onset of fatigue is still being elucidated, as the definition of fatigue is multidimensional and therefore different treatments may impact the development of the symptom via many routes (Karshikoff et al., 2017). While cancer treatments in general appear to cause fatigue, those undergoing treatment for brain cancer may experience further additional causes of fatigue related to changes in the sleep-wake regulation and masking. Researchers in neuro-oncology suggested that fatigue is a form of daytime sleepiness called hypersomnolence (Armstrong et al., 2017). Treatment-related hypersomnolence within the brain tumor population is associated with radiation therapy that targets the brain (Powell et al., 2011; Khan et al., 2018). Clearly, patients experiencing hypersomnia because of radiation are unable to maintain wakefulness during the daytime, which suggests a faltering of the masking pathway’s ability to suppress sleep urges in the light. As treatment in these patients is directed to the brain, we have hypothesized that changes in the neuroanatomy of sleep circuits may be directly impacted by therapy, including the circadian visual pathway that brings light information to the circadian and masking systems (Young et al., 2019; Shuboni-Mulligan et al., 2021).

This idea is further bolstered by two studies that examined the alteration in secreted melatonin after exposure to radiation in mice (Kassayova et al., 1999) and humans (Armstrong et al., 2017). Armstrong et al. (2017) suggested that patients experiencing sleep issues post-radiotherapy experienced heightened daytime melatonin levels. Melatonin is known as the hormone of darkness, as it is secreted during the night and production is actively suppressed with light exposure (Utiger, 1992). Light information to the pineal gland, where the majority of circulating melatonin is synthesized (Tan et al., 2018), is signaled through the circadian visual pathway (Lockley et al., 1997; Schwartz et al., 2009; Amaral and Cipolla-Neto, 2018); again, suggesting that this pathway, key for light-induced masking behavior, may be damaged during treatment in brain tumor patients leading to dysfunction of hormone regulation and sleep behavior. Further studies in the brain tumor population are merited, as these were small studies not designed to examine melatonin secretion specifically.

In patients with non-central nervous system (CNS) cancers, sleep disturbances have been shown to be associated with lower levels of this nighttime hormone, melatonin (Schernhammer and Schulmeister, 2004; Li et al., 2019). Melatonin in diurnal primates and humans is known to have a somnolence effect; when melatonin is given to subjects exogenously, body temperature is decreased immediately while there is also an increased sleepiness (Dollins et al., 1994; Zhdanova et al., 1995). We had previously proposed this as a masking effect that is separate from the circadian drive (Shuboni et al., 2016); additionally, melatonin has also been suggested to help mediate the light-induced masking response (Quay, 1970; Vilaplana et al., 1994; Burgess et al., 2001). In cancer patients that lack melatonin, issues with nighttime sleep maintenance would also be expected to be similar to individuals with removed pineal glands (Krieg et al., 2012; Slawik et al., 2016) or spinal injuries that prevent hormone secretion (Scheer et al., 2006; Whelan et al., 2020). Exogenous administration of melatonin as a method to prevent fatigue and overcome sleep issues has been proposed in patients with non-CNS cancers, with positive results in small sample studies (Innominato et al., 2015b) and clinical trials (Palmer et al., 2020; Sedighi Pashaki et al., 2021). The use of melatonin in the treatment of brain tumor patients undergoing radiotherapy, however, should be considered closely as some have proposed the hormone as a radiosensitizer, which are agents that enhance the lethal effects of radiation (Farhood et al., 2019). Both light and melatonin are agents that produce a clear immediate masking response to the presentation and are avenues by which sleep issues in patients can be treated, causing immediate relief, and will feed back into the circadian system to help strengthen entrainment of the master clock.

## Aging Effects of Masking

Age is an important factor in the expression of sleep and activity (Dijk et al., 1999, 2000; Mander et al., 2017). When homeostatic sleep or circadian rhythms are disrupted in older individuals there can be detrimental effects on survival (Tafaro et al., 2007; Davidson et al., 2008; Smagula et al., 2016; Hou et al., 2020). Within these systems, biological age impacts the physiological integrity of the structures important for regulating behavior (Pyrkov and Fedichev, 2019; Elliott et al., 2021). In the SCN, in particular, plasticity has been shown to decrease between the cells of the nuclei resulting in dampened circadian rhythms of behavior in older animals (Van Someren et al., 2002; Nakamura et al., 2016). The masking process, too, is impacted by age, as older mice have been shown to recover from masking pulses to light faster, resulting in less impactful and shorter-lasting masking effects on behavior, and exhibit structural changes within retinal ipRGCs and the circadian visual system of the brain (Adhikari et al., 2015; Shuboni-Mulligan et al., 2021). The pupillary light reflex (PLR), which like masking is also dependent on the circadian visual system and ipRGCs, is negatively impacted by age, resulting in a decreased change in pupillary size after exposure to light in humans (Bitsios et al., 1996; Lobato-Rincón et al., 2014). Clearly, old age is detrimental for many biological processes and impacts all the components of the homeostatic sleep, circadian rhythms, and masking processes. Therefore, the elderly population is positioned to develop sleep disturbances caused by disease. This further emphasizes the need to align sleep with the proper phase of the pacemaker and to obtain as much natural light during the day and to avoid light sources at night to keep masking in synchrony with these other systems.

## Conclusion

Environmental cues (e.g., light-dark cycle, temperature, food, socialization, drug usage) have an immediate and direct effect on behavior–a process called “masking.” Masking is capable of complementing entrainment if the cues align with the circadian system (e.g., in diurnal mammals, if lights are on during the day when the SCN is active), but can also work independently of entrainment (e.g., in diurnal mammals, if lights are on at night when the SCN is inactive). Similarly, masking is capable of complementing homeostatic sleep processes if the cues align with sleep (e.g., in diurnal mammals, if lights are on when the organism is awake, alert, and active during the day), but can also work independently of the homeostatic system (e.g., in diurnal mammals, if the lights are on when the organism is trying to sleep at night). Therefore, we argue in this review that masking is capable of complementing the circadian and homeostatic systems when synchrony and alignment between environmental cues is achieved (Figure 1), which is adaptive for the organism, but when these cues are misaligned with one or more of these systems, the systems are at odds promoting opposite behaviors, which is maladaptive for the organism. It should be noted that the type of environmental stimulus, time-of-day when it is presented, and the temporal niche of the organism should all be considered, as different effects occur in each circumstance.

In this review, we have discussed the importance of masking, highlighting the critical need for masking to complement the circadian and homeostatic systems in order to result in improved health. We have also considered the important role of development, and how aging is associated with dampening of the circadian, homeostatic, and masking systems. But again, when these three systems are synchronized with one another, they are capable of supporting one another resulting in benefits to health. We hypothesize that when elderly humans get as much natural light as possible during the day, socialize during the day, live in warmer ambient temperatures during the day (and all of these things opposite at night), the circadian, homeostatic, and masking systems will be in alignment resulting in improved sleep quality and quantity, therefore improving health overall. Altogether, when done at the appropriate time-of-day, masking can be used as a non-invasive treatment option to help prevent suffering from sleep-related disorders.

## Author Contributions

Both authors listed have made a substantial, direct, and intellectual contribution to the work, and approved it for publication.

## Conflict of Interest

The authors declare that the research was conducted in the absence of any commercial or financial relationships that could be construed as a potential conflict of interest.

## Publisher’s Note

All claims expressed in this article are solely those of the authors and do not necessarily represent those of their affiliated organizations, or those of the publisher, the editors and the reviewers. Any product that may be evaluated in this article, or claim that may be made by its manufacturer, is not guaranteed or endorsed by the publisher.
